# Endoluminal Vacuum Therapy Using a New “Fistula Sponge” in Treating Defects of the Upper Gastrointestinal Tract—A Comparative, Retrospective Cohort Study

**DOI:** 10.3390/medicina60071105

**Published:** 2024-07-07

**Authors:** Florian Richter, Claudio Conrad, Julia Hoffmann, Benedikt Reichert, Witigo von Schoenfels, Clemens Schafmayer, Jan-Hendrik Egberts, Thomas Becker, Mark Ellrichmann

**Affiliations:** 1 Department of General, Visceral-, Thoracic-, Transplantation- and Paediatric Surgery, University Medical Center Schleswig-Holstein (UKSH), Campus Kiel, 24105 Kiel, Germany; florian.richter@uksh.de (F.R.); j.hoffmann@uksh.de (J.H.); benedikt.reichert@uksh.de (B.R.); witigo.vonschoenfels@uksh.de (W.v.S.); thomas.becker@uksh.de (T.B.); 2 Department of Internal Medicine I, University Medical Centre Schleswig-Holstein (UKSH), Campus Kiel, 24105 Kiel, Germany; claudiocim.conrad@uksh.de; 3 Department of General Surgery, University Hospital Rostock, 18057 Rostock, Germany; clemens.schafmayer@med.uni-rostock.de; 4 Department of Surgery, Israelitisches Krankenhaus, 22297 Hamburg, Germany; j.egberts@ik-h.de

**Keywords:** fistula sponge, Eso-Sponge^®^, anastomotic insufficiencies, esophageal perforation, endoscopic treatment

## Abstract

*Background and Objectives*: Anastomotic insufficiencies (AI) and perforations of the upper gastrointestinal tract (uGIT) result in high morbidity and mortality. Endoscopic stent placement and endoluminal vacuum therapy (EVT) have been established as surgical revision treatment options. The Eso-Sponge^®^ is the only licensed EVT system with limitations in treating small defects (<10 mm). Therefore, a fistula sponge (FS) was developed for the treatment of such defects as a new therapeutic approach. The aim of this study was to evaluate both EVT options’ indications, success rates, and complications in a retrospective, comparative approach. *Materials and Methods*: Between 01/2018 and 01/2021, the clinical data of patients undergoing FS-EVT or conventional EVT (cEVT; Eso-Sponge^®^, Braun Melsungen, Melsungen, Germany) due to AI/perforation of the uGIT were recorded. Indication, diameter of leakage, therapeutic success, and complications during the procedure were assessed. FSs were prepared using a nasogastric tube and a porous drainage film (Suprasorb^®^ CNP, Lohmann & Rauscher, Rengsdorf, Germany) sutured to the distal tip. *Results*: A total of 72 patients were included (20 FS-EVT; 52 cEVT). FS-EVT was performed in 60% suffering from AI (cEVT = 68%) and 40% from perforation (cEVT = 32%; *p* > 0.05). FS-EVT’s duration was significantly shorter than cEVT (7.6 ± 12.0 d vs. 15.1 ± 14.3 d; *p* = 0.014). The mean diameter of the defect was 9 mm in the FS-EVT group compared to 24 mm in cEVT (*p* < 0.001). Therapeutic success was achieved in 90% (FS-EVT) and 91% (cEVT; *p* > 0.05). *Conclusions*: EVT comprises an efficient treatment option for transmural defects of the uGIT. In daily clinical practice, fistulas < 10 mm with large abscess formations poses a special challenge since intraluminal cEVT usually is ineffective. In these cases, the concept of extraluminal FS placement is safe and effective.

## 1. Introduction

Esophageal perforations and anastomotic insufficiencies (AI) present significant therapeutic challenges due to their high rates of serious complications, such as mediastinitis and sepsis, which can result in mortality rates ranging from 5% to 50% [1,2,3,4,5,6]. These perforations may arise from ulceration, circulatory disorders, carcinoma, violent vomiting, or medical interventions [7]. Iatrogenic perforations are the most common, comprising 59% to 63% of cases [1,2]. The mortality rate of esophageal perforations is strongly influenced by the location of the perforation and its proximity to the mediastinum. Consequently, thoracic esophageal perforations result in higher mortality rates of 5.4% to 36%, compared to cervical perforations at 6% to 20%, and abdominal perforations at 3% to 22% [8,9,10,11,12].

Operations in the upper gastrointestinal tract (uGIT) carry a significant risk of AI in 2% to 25% of cases, often resulting in spillage of fluids or food particles that lead to inflammation of the surrounding tissue. The surgical technique, the level of the anastomosis, and any neoadjuvant treatments play a crucial role in this context [13,14]. AI-associated mortality in the uGIT can reach up to 67%, which is higher compared to esophageal perforations [15,16]. Given the exceptionally high mortality rates, prompt and appropriate treatment is essential for managing transmural defects of the uGIT. Treatment options include conservative measures, surgical debridement, closure of the dehiscence, or re-attachment of an anastomosis with simultaneous drainage [17]. In addition to surgical revision, various endoscopic interventions, such as clip closure, fibrin glue injection, endoluminal drainage via nasogastric tubes, endoluminal suturing, and the implantation of self-expanding fully covered metal stents (SEMS), are employed [18,19]. Furthermore, endoluminal vacuum therapy (EVT) has been successfully established in the management of these defects. Several studies have demonstrated a remarkably high recovery rate of approximately 90%, with an associated mortality rate of only 10% following EVT [20,21].

The concept of EVT is based on the principles of negative pressure wound therapy, which creates a controlled sub-atmospheric environment that enhances tissue perfusion, reduces edema, and stimulates the formation of granulation tissue. This environment induces microdeformation at the cellular level, encouraging cell proliferation and migration, which are essential for tissue repair and regeneration. Macrodeformation helps to approximate the wound edges and reduce the size of the defect. Continuous suction removes exudate and reduces the risk of infection, maintaining a clean wound environment conducive to healing [20,21].

Placing the sponge, especially when it needs to be inserted extraluminally into a wound cavity, can be technically challenging or even impossible in some clinical situations. In these complex scenarios and in the treatment of very small defects with a diameter of less than 10 mm, a specialized, self-designed fistula sponge (FS) has been incorporated into our department’s treatment algorithm. The FS consists of a porous drainage foil sutured to the distal tip of a nasogastric tube (Suprasorb^®^ CNP Wound Foam, Lohmann & Rauscher GmbH & Co. KG, Neuwied, Germany). This fistula sponge can be inserted under direct visual control using either grasper forceps, a guide wire, or a combination of both.

So far, the use of FS has only been reported in small case series but comparative evaluations of both EVT concepts are lacking [22]. Therefore, this study aimed to evaluate indications, technical and clinical success rates, and complications of both EVT systems (cEVT and FS) in a retrospective, comparative manner.

## 2. Materials and Methods

### 2.1. Study Design

All patients who were treated with EVT due to AI or perforation of the uGIT at the University Medical Center Schleswig-Holstein, Campus Kiel, Kiel, Germany between September 2018 and January 2021 were included in this retrospective, comparative cohort study.

After an interdisciplinary discussion of each case, EVT was initiated with either FS or cEVT using the Eso-SPONGE^®^-System (B.Braun, Aesculap AG, Braun Melsungen, Germany) in close collaboration of the Department of Interdisciplinary Endoscopy and the Department of General, Visceral, Thoracic, Transplantation and Pediatric Surgery as a standard therapeutic approach of daily clinical practice. Baseline characteristics and clinical criteria were evaluated during clinical follow up as standard procedure.

**Figure 1:**
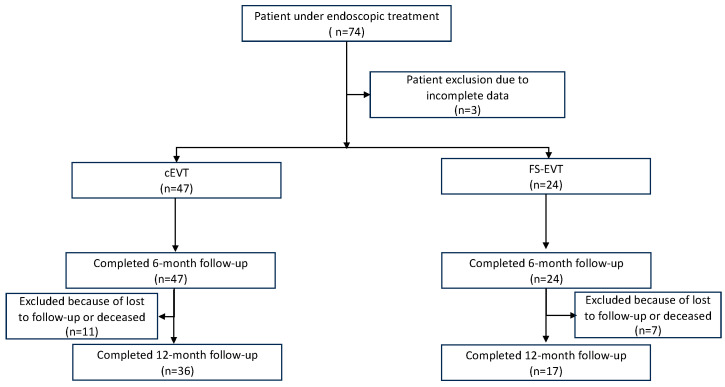
Flowchart of the inclusion of the study. cEVT: conventional endoluminal vacuum therapy; FS-EVT: fistula sponge–endoluminal vacuum therapy.

The flowchart of this study is summarized in Figure 1.

This study was reviewed by the ethics committee of the Medical Faculty of Christian Albrecht University. There were no professional ethical or legal objections to conducting the study (license number: A141/14).

Prior to the selection of patients, the following eligibility criteria were specified:

### 2.2. Inclusion Criteria

- Transmural wall defect of the uGIT.
- Independent indication for endoscopic vacuum therapy after interdisciplinary case discussion.
- Valid declaration of consent from the UKSH for scientific data collection (“broad consent” UKSH), in the electronic patient file.

### 2.3. Exclusion Criteria

- Deviation from the standard procedure of EVT.

### 2.4. Endpoints

- The primary endpoint was defined as the clinical success, namely the complete resolution of the transmural defect lined with granulation tissue.
- Secondary endpoints were: patient characteristics, duration of EVT, number of changes of respective EVT-system, the initial size of transmural defect, complications, and rate of morbidity and mortality during follow-up.

### 2.5. Conventional Endoluminal Vacuum Therapy (cEVT)

**Figure 2:**
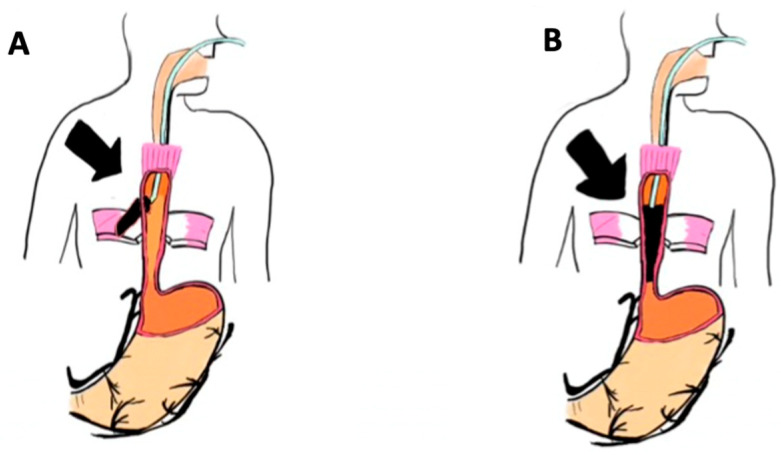
Schematic overview of conventional endoluminal vacuum therapy: (**A**) Intracavital/extraluminal implantation of conventional endoluminal vacuum therapy; (**B**) endoluminal placement of conventional endoluminal vacuum therapy (picture created by JH). Black arrow marks the transmural defect of the esophageal wall.

The Eso-SPONGE^®^-therapy was performed as described in a previous publication [23]. Briefly, after endoscopic evaluation of the transmural defect, the cEVT-system was adapted to the size of the respective defect and then inserted extraluminally into the cavity. Based on the size of the defect, EVT was carried out either by endoscopic insertion of an Eso-Sponge^®^ into the abscess cavity extraluminal placement, or, in case of a limited defect size and the absence of a cavity, by endoscopic insertion into the lumen of the esophagus itself (Figure 2). With diminution of the defect over the course of therapy, the sponge placement could be moved from its initial intracavitary/extraluminal location (Figure 2A) to an intraluminal/endoesophageal position (Figure 2B) allowing for complete cure of a potential residual leakage.

### 2.6. Fistula Sponge (FS) Therapy

For FS therapy, a layered compressed porous drainage foil (Suprasorb^®^ CNP Wound Foam, Lohmann & Rauscher GmbH & Co. KG, Neuwied, Germany) was sutured to the distal end of a nasogastric tube (diameter between 8 and 12 Charriere) at a length of 1–6 cm depending on the depth of the defect in two to five wraps by adjusting the FS-EVT to the diameter of the defect. The suture was composed of a non-absorbable braided Mersilene^®^ thread of strength 2–0 (Ethicon, Somerville, NJ, USA) every 5 mm along the entire length of the drainage foil. This fistula sponge was then inserted endoscopically into the target area using a grasping forceps and/or a guidewire (Jagwire 0.035’, Boston Scientific, Malborough, MA, USA) on the endoscopist’s request.

The commercially available Eso-SPONGE^®^ and the newly created fistula sponge are shown in Figure 2 in direct comparison. The respective EVT system was then diverted transnasally and connected to a vacuum pump (Smith & Nephew plc, Watford, Hertfordshire, UK) with continuous suction of 120 mmHg. Changes to the EVT system were scheduled approximately every 3 days until complete resolution of the cavity (as defined) was achieved. Before removing the respective sponge system, both systems were disconnected and flushed with 20 mL of saline 0.9% to prevent the systems from tearing off due to the granulation tissue growing into the pores. The location of the transmural defect was assessed endoscopically in centimeters (cm) from the dental arch (DA); the diameter and length were measured using a guidewire and/or biopsy forceps. Data obtained from endoscopy were compared to results from computer tomography (CT) scan. The different locations of the defects were categorized into four sections: (1) 15–23 cm DA, (2) 24–32 cm DA, (3) 33–40 cm DA, and (4) >40 cm DA.

### 2.7. Data Collection and Follow-Up

All clinically relevant data were obtained by our hospital information system (Orbis, Dedalus, Bonn, Germany). Patients received a routine follow-up after 30 days and 6 and 12 months after discharge. Follow up information were collected by our outpatient clinic or respective hospital readmissions.

### 2.8. Bench Study to Compare Throughput of EVT Systems

**Figure 3:**
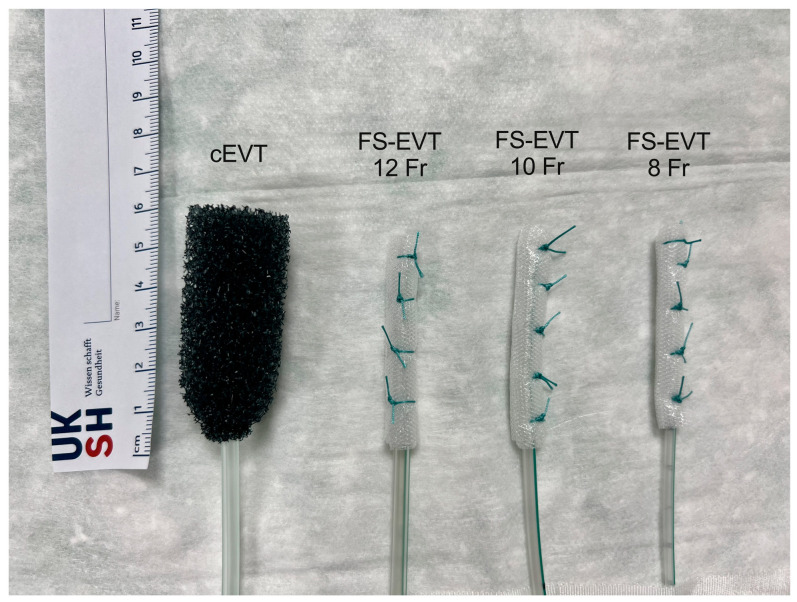
Comparison of the systems for endoluminal vacuum therapy; cEVT (Eso-SPONGE^®^) and FS-EVT with diameters of 12 Fr/10 Fr/8 Fr (from left to right). cEVT: conventional endoluminal vacuum therapy; Fr: French; FS-EVT: fistulassponge–endoluminal vacuum therapy (picture created by ME).

To compare the suction capacity of the two EVT options in a standardized way, volume measurements of liquid sucked out of a vessel by the respective sponge at defined periods of time were recorded (Figure 3). To compare the suction performance of the two EVT options in a standardized manner, the FS-EVT or the cEVT system were dipped into a container with 2 L of drinking water and connected to the mentioned vacuum pump with continuous suction of 120 mmHg simulating the clinical situation in patients. The volume of water drawn from the container was measured after 30, 60, 90, and 120 s. Measurements were repeated five times each to minimize fluctuation-induced errors in the readings.

For the cEVT, three sizes of the Eso-SPONGE^®^ were tested: complete Eso-SPONGE^®^ 13 mm, one half and one third of the respective sponge.

For the FS-EVT, a nasogastric tube of 12 Ch was used, having five wraps of drainage foil at the distal tip over a length of 5, 2.5, and 1.4 cm (length of 100%, 50%, 30%) corresponding to the respective lengths of the cut Eso-SPONGE^®^ 13 mm.

### 2.9. Statistical Analysis Methods

Descriptive statistics of patient characteristics and treatment details were calculated using mean values with standard deviation (SD). Statistical analyses of mean values and SD, such as age, BMI, time intervals, defect sizes, number of changes, and suction capacities, were carried out using unpaired *t*-tests. Categorical comparisons and rates were assessed using Fisher’s exact tests. The significance level was set at a *p*-value of <0.05. All statistical analyses and graphical processing were carried out using GraphPad Prism software, version 8.0 (GraphPad Software, San Diego, CA, USA). Patients with missing relevant data were excluded from further analyses.

## 3. Results

### 3.1. Patient Characteristics

**Figure 4:**
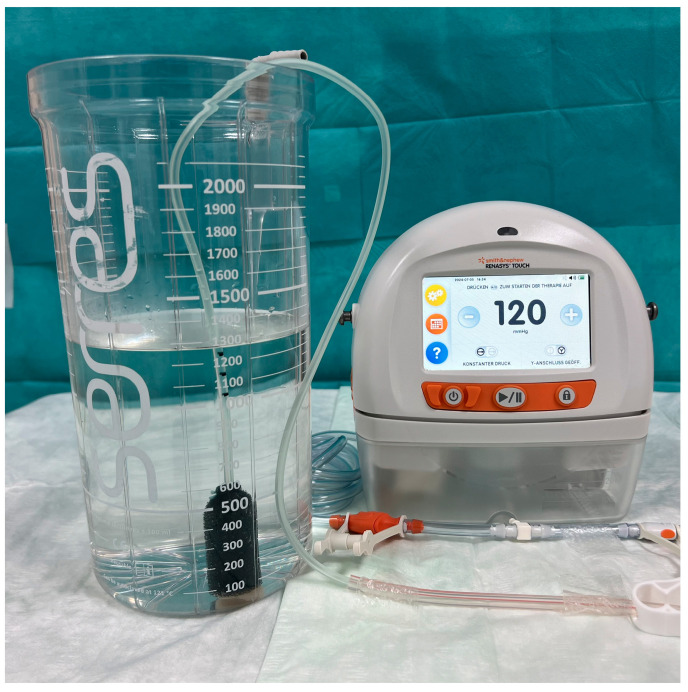
Experimental set-up for comparative measurement of throughput of the two endoluminal vacuum therapy systems (picture created by ME).

A total of 71 patients (13 females, 31.7%) was evaluated in this study, among these 47 (66.2%) were treated with cEVT and 24 (33.8%) with FS-EVT (Figure 4). Further details of patient characteristics are summarized in Table 1.

### 3.2. Primary EndpointRate of Defect Healing

**Figure 5:**
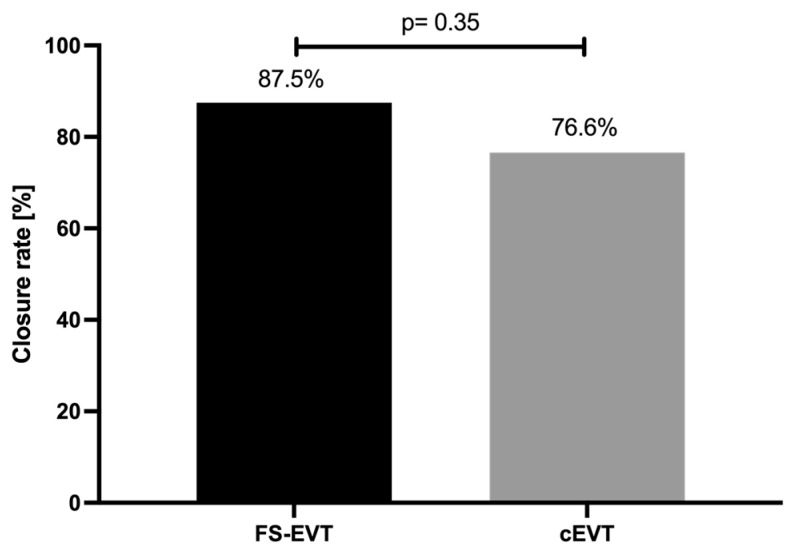
Comparison of closure rates of conventional endoluminal vacuum therapy vs. fistula sponge–endoluminal vacuum therapy. cEVT: conventional endoluminal vacuum therapy; FS-EVT: fistula sponge–endoluminal vacuum therapy.

As previously mentioned, closure rate of the transmural defects in the uGIT was defined as the primary endpoint. Patients receiving cEVT experienced a complete defect closure in 76.6% of cases, whereas FS-EVT resulted in a closure rate of 87.5%; the difference in the rate of success was statistically not significant between these groups (*p* = 0.355; Figure 5).

### 3.3. Secondary Endpoint

#### 3.3.1. Defect Size

**Figure 6:**
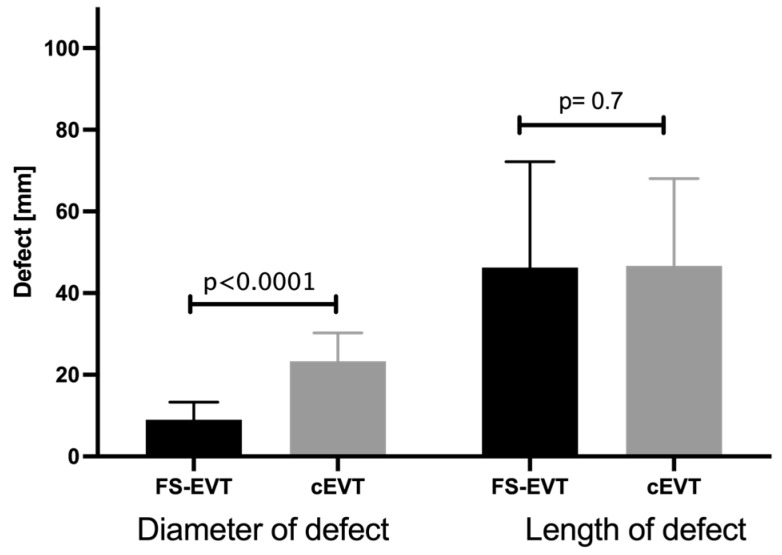
Comparison of diameter and length of the wall defects between the FS-EVT and cEVT group (cEVT: conventional endoluminal vacuum therapy; FS-EVT: fistula sponge–endoluminal vacuum therapy).

Based on endoscopic and radiological evaluation, defect sizes were quantified in each patient as length and entrance diameter of the respective defect. The mean diameter of the wall defect of 9.0 ± 4.3 mm in the FS-EVT group differed significantly from the diameter of 23.3 ± 6.9 mm in the cEVT group (*p* < 0.0001). The mean length of the lesions did not show a statistical difference between the two collectives (FS-EVT = 46.3 ± 25.9 mm; cEVT = 46.7 ± 21.4 mm; *p* = 0.7; Figure 6).

#### 3.3.2. Duration of Therapy and Number of Sponge Changes

**Figure 7:**
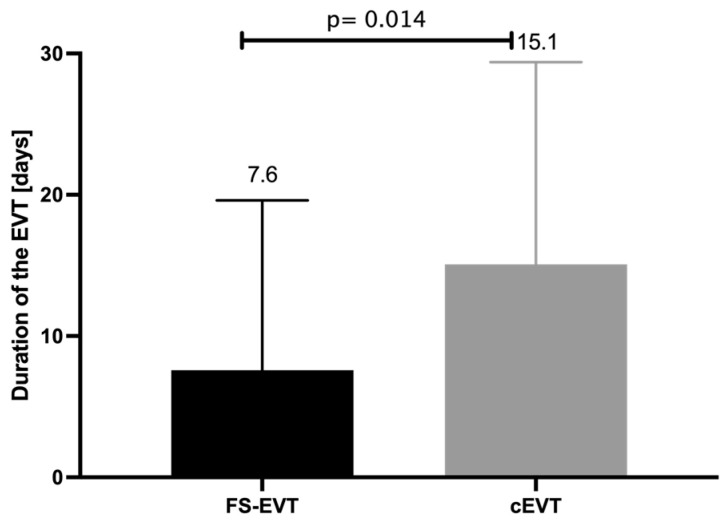
Comparison of duration of EVT of FS-EVT and cEVT group in days (cEVT: conventional endoluminal vacuum therapy, FS-EVT: fistula sponge–endoluminal vacuum therapy).

While the average treatment period in the cEVT group was for 15.1 ± 14.3 days, the average treatment period in the FS-EVT group was significantly shorter with only 7.6 ± 12.0 days (*p* = 0.014, Figure 7).

**Figure 8:**
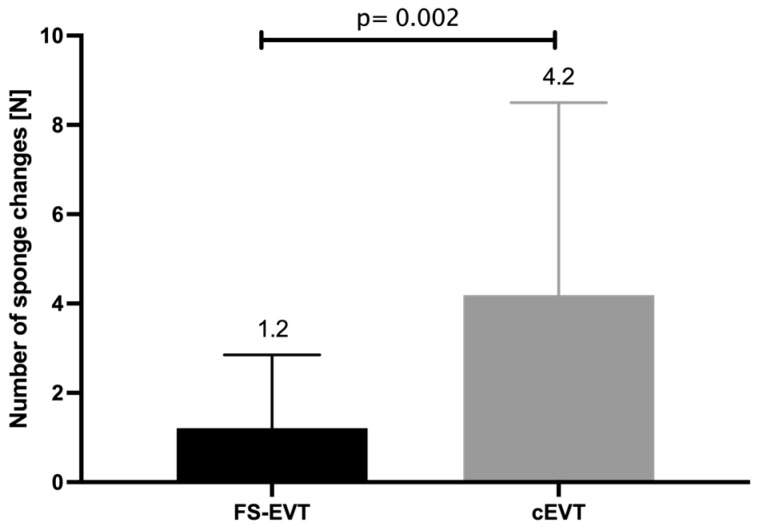
Comparison of number of sponge changes of both groups (cEVT: conventional endoluminal vacuum therapy, FS-EVT: fistula sponge–endoluminal vacuum therapy.

During the respective treatment interval, EVT systems were exchanged 1.2 ± 1.6 times in the FS-EVT group compared to 4.2 ± 4.3 times in the cEVT group (*p* = 0.002, Figure 8).

#### 3.3.3. Time Interval until the Start of the EVT

For the evaluation of the time interval between initial diagnosis and initiation of EVT, only patients with AI as a result of surgery were considered. All patients with an acute perforation of the uGIT during an endoscopic intervention were immediately treated with EVT in the same session. For the AI group, despite a difference of 6.4 days, the time interval until the start of vacuum therapy did not differ significantly (*p* = 0.255) between the cEVT group (5.8 ± 5.68 d) and the FS group (12.2 ± 22 d).

#### 3.3.4. Complication Rate and Therapy

The total complication rate was 27.7% (13/47) in the cEVT group and 20.8% (5/24) in the FS-EVT group (*p* = 0.6). Complications observed during ongoing therapy or sponge exchange procedures, such as sponge ingrowth into the mucosa and subsequent tearing during removal, did not occur in either group. In one patient, hemorrhage occurred during ongoing therapy, which contributed to the patient’s death. Placement of the fistula sponge was challenging in two patients: in one case, due to non-compliance with repeated self-removal by the patient, and in the other case, due to repeated dislocation caused by anatomical conditions.

Complications, such as persistent or recurrent fistulas and esophageal stenoses in the area of the previous EVT, were primarily detected during follow-up examinations. In the cEVT group, eight patients developed stenoses over the long term. With the exception of one stenosis caused by tumor recurrence, these were successfully treated with balloon dilation. In the FS group, three patients developed stenoses, all of whom were also successfully treated with balloon dilation. Persistent fistulas or fistula recurrences occurred in four patients in the cEVT group, with none occurring in the FS-EVT group. Three patients in the cEVT group underwent repeat EVT, and one patient with an extensive esophagotracheal fistula required surgery. Further details of patient complications are summarized in Table 2.

#### 3.3.5. Follow-Up, 30-Day Mortality

**Figure 9:**
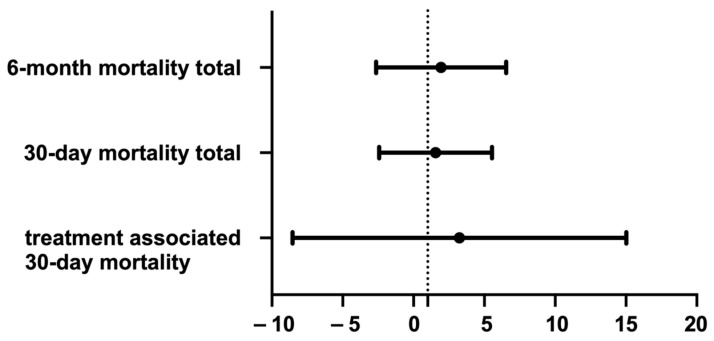
Odds ratio comparing 30-day mortality in total and treatment associated, as well as 6-month mortality total.

In the control group a total of 10 patients (21.3%) died within 30 days after the initial event (perforation or surgery) compared to 5 patients (20.8%) in the FS-EVT group. A subgroup analysis revealed an EVT-associated 30 d mortality of 4.3% (cEVT) and 4.0% (FS-EVT) (*p* = 0.9; Table 3; Figure 9).

#### 3.3.6. Bench Testing of Suction Capacity

**Figure 10:**
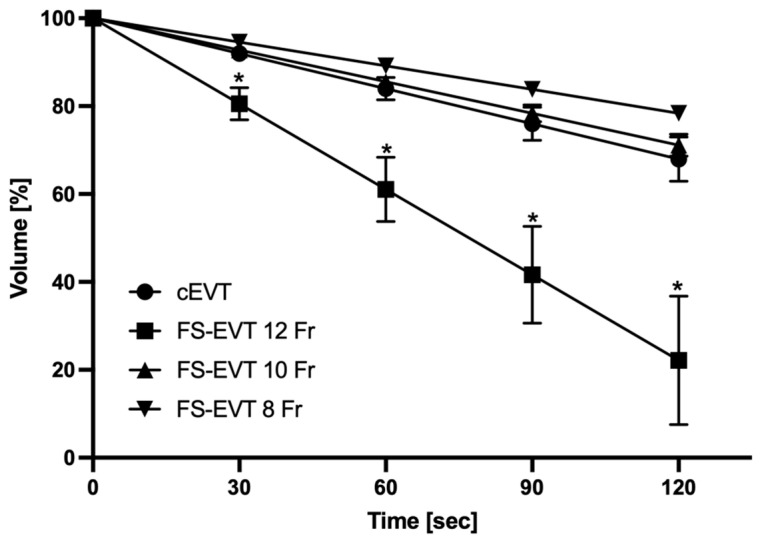
Comparison of suction capacity mL/30 s. Fistula sponge–endoluminal vacuum therapy 12 Fr showed a significantly improved suction capacity (*: *p* = 0.01). cEVT: conventional endoluminal vacuum therapy; Fr: French; FS-EVT: fistula sponge–endoluminal vacuum therapy.

Suction capacity was quantified in mL per 30 s. The cEVT system showed a constant suction capacity of 60 mL/30 s; no differences were observed comparing the different lengths of the cEVT. FS-EVT with a 12 French (Fr) gastric tube resulted in a significantly increased suction capacity of 92 mL/30 s (*p* = 0.002) independent of the length. The other FS-EVT tests with a 10 Fr and 8 Fr gastric tube revealed significantly lower suction levels compared to the 12 Fr system (10 Fr = 72 mL/30 s; 8 Fr = 43 mL/30 s). These amounts were comparable to the cEVT (Figure 10).

## 4. Discussion

Esophageal perforation and AI following esophageal resections can lead to serious complications resulting in high morbidity and mortality of the patients [24,25]. For a long time, the only available therapeutic options for perforations were either endoscopic stent placement or surgery [26].

However, several stent-associated complications, such as dislocation, penetration into the wall layers of the digestive tract and formation of abscesses, are reported in the present literature in 13–21% of patients with the recurrent need of further endoscopic or surgical intervention [27,28].

Since 2007, the technique of EVT has been successfully approved for the treatment of AI and perforations of the uGIT [20,29,30]. In 2013, our group conducted a retrospective comparison of mortality rates in patients treated with surgical revision, EVT, or SEMS placement for defects of the upper gastrointestinal tract following oncologic surgery. After adjusting for the APACHE II score, we found that patients treated with surgical revision and those with SEMS placement had significantly higher mortality rates compared to those treated with EVT. A limitation of our analysis was the lack of comparison of closure rates and the timing of treatment post-defect diagnosis, which may introduce potential bias. Nonetheless, these findings initiated a paradigm shift in the management of postoperative defects, at least in our center [21].

A limitation in clinical practice is that commercially available sponges are not well-suited for complex anatomic situations or for small defects or fistulas with a diameter of less than 10 mm. To address this, a special fistula sponge, consisting of a gastric tube wrapped with a porous foil and secured by sutures (Suprasorb^®^ CNP Wound Foam, Lohmann & Rauscher GmbH & Co. KG, Neuwied, Germany), was first described by Loske et al. [31]. This custom-designed sponge has been further adjusted and routinely used in our center since 2018.

So far, the available data evaluating this new technique are very limited and do not assess criteria for patient selection and short- and long-term outcomes. In the initial publication, the concept of FS-EVT was applied to a heterogeneous cohort of eleven patients with duodenal leakages resulting from either acute perforation or anastomotic insufficiency (AI). The two EVT methods were used either individually, in combination, or consecutively, leading to complete closure of the defect in all patients after a median therapy duration of 11 days (range 7–24 days) [22]. In a small case series involving nine patients with iatrogenic esophageal defects, EVT, whether as cEVT or FS-EVT, was successfully applied in 100% of the reported cases after a median treatment duration of 19 days [32].

Based on these studies, criteria for patient selection for the respective EVT are not defined. Prior to initiation of EVT, the following patient criteria should be evaluated to design the optimal treatment strategy in an interdisciplinary approach: patient’s general condition, level of infection parameters, diameter and length of the wall defect, presence of an extraluminal wound cavity, as well as radiological findings.

In our study, a diameter of the wall defect of <10 mm was defined as cut-off to initiate FS-EVT as first treatment option. Other research groups have treated defects with a size < 15 mm with FS-EVT either from the beginning or during the course of therapy after previous cEVT [22,32]. According to Kaczmarek et al., defects with a diameter of less than 10 mm can be either treated with FS-EVT or initial balloon dilation followed by cEVT [33]. However, our center does not utilize this latter approach to reduce local trauma to the esophageal wall and adjacent wound cavity. Based on the high success rates demonstrated by the FS-EVT concept in the presented data, we cannot recommend this approach.

In addition to the aforementioned case series, several case reports have demonstrated the efficacy of EVT with open-pore drainage film in various anatomical locations and pathologies. Wulfert et al. documented the successful treatment of peritonitis following a complicated cesarean section using intrauterine vacuum therapy. Combined with abdominal lavage, FS-EVT controlled the septic focus within eleven days, thus preventing the need for hysterectomy [34]. Loske et al. reported a case where a significant defect in the urinary bladder occurred during a rectal amputation. Continuous vacuum therapy, in conjunction with ureteral stenting, resulted in complete closure of the bladder wall defect after 18 days [35].

All of the published case series reflected a high therapeutic success of 100% of the FS-EVT concept. In our current study, we observed a mean closure rate of 82.1% (cEVT = 76.6%; FS-EVT = 87.5%). These findings are consistent with a multicenter cohort study conducted by our group on cEVT for esophageal wall defects, which included a total of 102 patients and demonstrated a complete closure rate of 86.3%. Within this cohort, the closure rates for anastomotic insufficiency (AI) and acute perforation were 91.3% and 75.8%, respectively [36].

It is important to note that the criteria for defining complete wound healing to terminate EVT vary significantly among different centers and case series. While some groups terminate vacuum therapy after observing a reduction in the cleaned wound cavity and the formation of granulation tissue with normalized serum infection parameters [37], our study, supported by the case series of Wichmann et al. [32], defined defect healing as the presence of clean granulation tissue in the wound bed without persistent fistula. This was verified through endoscopic examination, X-ray imaging with contrast medium, and ensuring the residual cavity depth was less than 10 mm.

Despite the high success rate and ease of implementation, the EVT-associated complications, such as ingrowth of the sponge into the mucosa, dislocation of the sponge, bleeding, subsequent stenosis of the uGIT or the development of a fistula recurrence in the course of treatment, need to be mentioned [38,39,40,41,42].

We observed an overall complication rate of 24.3%, with no significant differences between the intervention groups (cEVT = 27.7%; FS-EVT = 20.8%). Except for one patient, only minor complications occurred as previously described. In the cEVT group, one patient experienced hemodynamically significant bleeding during the course of therapy, resulting in death. This complication, known to occur in up to 10% of cEVT patients, arises from the erosion of feeding vessels within the wound cavity [23,41]. Jung et al. reported only minor bleedings, potentially related to significantly lower suction rates of −20 to −50 mmHg [43]. In general, due to the smaller pores of the drainage foil compared to cEVT, the risk of granulation tissue ingrowth into the FS-EVT system and subsequent significant bleeding appears to be lower. However, this assumption cannot be reliably confirmed based on the current literature, including our study [44]. To mitigate the risk of significant bleeding complications, it is recommended to consider reducing suction force, performing mandatory pre-interventional CT scans, and preferentially using foil-based suction systems in high-risk patients.

Eight of our patients (17%) in the cEVT group developed esophageal strictures during follow-up that could all be successfully treated by endoscopic balloon dilation. In the FS-EVT group, strictures occurred in three patients (12%) with the same consecutive therapeutic approach and resolution rate. These data stand in line with other publications that presented scarring stenoses as long-term complications in 4.2–18.5% of treatments for AI or perforations in the uGIT [23,39,43].

In our cohort, we observed a treatment-associated 30-day mortality rate of 4.3% in the cEVT group and 4.0% in the FS-EVT group. The other patients contributing to the relatively high overall mortality rate experienced either postoperative multi-organ failure occurring immediately after surgery or progression of the underlying oncological disease. These treatment-associated mortality rates are comparable to the reported rates of 6.7% to 12.9% [39,43,45]. The aforementioned multicenter study conducted by our working group analyzed data from eleven hospitals and included a total of >100 patients who received EVT for the treatment of a wall defect in uGIT. The results published in this study are consistent with mortality rates of approximately 6.7% [36].

Two alternative concepts are discussed in the literature for the treatment of small transmural defects and fistulas: (i) endoscopic internal drainage (EID) and more recently (ii) vacuum stents combining the EVT concept with an SEMS.

In the EID concept, a pigtail drainage catheter is endoscopically placed in the fistula and adjacent cavity, facilitating passive drainage of purulent material and promoting wound granulation through continuous mechanical irrigation of the cavity [46]. A recent review and meta-analysis by Laopemathong et al. compared the effectiveness of EVT and EID for treating post-bariatric leaks. The study found that EVT had a slightly lower healing success rate of 85.2% compared to 91.6% for EID when used as a first-line treatment [47]. Similarly, a study by Jung et al. comparing EID and EVT in patients with defects following oncologic surgery reported a higher healing success rate of 100% for EID, compared to 85.2% for EVT [48]. Potentially, FS-EVT combines the advantages of a small-diameter drainage system with the EVT concept, which could further enhance clinical outcomes in these complex situations. However, no studies are currently available that compare EID to FS-EVT.

A newly introduced vacuum stent (VacStent GI, Möller Medical, Fulda, and Microtech, Düsseldorf, Germany) features a fully-covered self-expanding metal stent with an external cylindrical polyurethane sponge. The application of this system is comparable to the placement of a conventional SEMS. Once in place, the stent allows for enteral nutrition during the course of EVT. Despite its potential for broad application, the clinical necessity of this system still needs to be established. A prospective registry study demonstrated that the vacuum stent effectively combines the benefits of stent therapy with EVT for uGIT perforation, AI, and as a preventive measure. Functional closure was observed in 70% of patients after vacuum stent placement, comparable to success rates of the cEVT. In combination with complementary therapies, the healing rate could be increased to approximately 80% [49,50]. Factors, such as the chronicity and location of the leak or fistula and the level of applied suction vacuum, influence the healing rate. It remains unclear whether this system is as effective as cEVT and FS-EVT for small-diameter leaks and fistulas with adjacent larger wound cavities. Due to the high heterogeneity of the recruited patients, no robust clinical recommendations can be made at this time, and prospective trials are needed comparing technical and clinical success rates, as well as cost effectiveness.

### Comparative Measurements of Suction Power

Since no data were available so far evaluating the suction capacity of the two EVT systems, we performed a bench study to accurately quantify the volume of aspirated fluid per time at intervals of 30 s. The combination of a 12 Fr gastric tube and the porous drainage foil especially resulted in significantly increased suction rate compared to the cEVT and to FS-EVT with 10 Fr and 8 Fr gastric tubes. Of note, the suction capacity is independent from the length of the EVT system but only correlates to the diameter of the drainage tube. These results confirm the observations described by other authors [31,32,44]. Due to a lower adherence to the wall, the film-based drainage ensures a more effective removal of fluids. The double-layered film with regularly arranged pores reliably drains secretions inwards via its interstitial space and thus apparently also qualifies this technique for prophylactic use after resections in the uGIT [51].

The number of patients with transmural wall lesions in the uGIT treated with endoscopic vacuum therapy and a customized fistula sponge composed of Suprasorb^®^ film investigated in our work is the largest collection published to date.

Nevertheless, the present study also has its own limitations. In the treatment of wall defects in the uGIT, different practitioners with different levels of experience were involved and accordingly influenced the respective course and outcomes of therapy. Furthermore, the entire study was designed as a retrospective observation of our cohort in daily clinical practice. Future prospective, randomized controlled trials are needed to finally define advantages and disadvantages of the different EVT techniques. Since we only investigated defects of the uGIT in a retrospective manner, the obtained results cannot be generalized to all defects in the whole GIT.

## 5. Conclusions

In conclusion, endoscopic vacuum therapy encompasses several outstanding treatment options for transmural lesions in the uGIT. These include the new extraluminal FS-EVT placement using a porous drainage film that represents a safe and effective strategy for treating small wall defects or defects that cannot be negotiated with the cEVT system. In an interdisciplinary setting, this minimally invasive organ-preserving technique is an essential part of our armamentarium for the treatment of potentially life-threatening wall defects of the uGIT.

## Figures and Tables

**Table 1:** Patient characteristics.

	cEVT	FS-EVT	*p*-Value
Number of patients [N]	47	24	
Age [years]	62.8 ± 15.7	65.0 ± 16.3	0.42
Female [N] (%)	11 (23.4)	2 (8.3)	0.19
Indication [N] (%)			
Anastomotic insufficiency	21 (44.7)	8 (33.3)	0.67
Perforation	26 (55.3)	16 (66.7)	0.64
Location of defect from dental arch [N] (%)			
15–23 cm	3 (6.4)	2 (8.3)	0.84
24–32 cm	30 (63.8)	14 (58.3)	0.78
33–40 cm	10 (21.3)	7 (29.2)	0.73
>40 cm	4 (8.5)	1 (4.2)	0.52
Body mass index [kg/m^2^] (mean ± SD ^1^)	27.6 ± 8.9	26.0 ± 6.4	0.58
ASA ^2^ Score	3 (2–4)	2 (1–4)	0.87
Malignancy [N] (%)	31 (65.9)	18 (75)	0.59
Neoadjuvant treatment [N] (%)	18 (38.3)	9 (37.5)	0.99
Interval between surgery and EVT [d] (mean ± SD)	5.8 ± 5.7	6.2 ± 5.5	0.84

^1^ SD: Standard deviation; ^2^ ASA: American Society of Anesthesiologists.

**Table 2:** EVT-associated complications.

EVT-Associated Complications	cEVT ^1^ (N = 47)	FS-EVT ^2^ (N = 24)	*p*-Value
Bleeding [N] (%)	1 (2.1)	0 (0)	>0.99
Ingrowth/Tearing off of the sponge [N] (%)	0 (0)	0 (0)	>0.99
Technically not possible [N] (%)	0 (0)	2 (8.3)	0.11
Stenosis [N] (%)	8 (17)	3 (12.5)	0.74
Fistula recurrence or persistent fistula [N] (%)	4 (8.5)	0 (0)	0.29
Total complication rate [N] (%)	13 (27.7)	5 (20.8)	0.58

^1^ cEVT: conventional endoluminal vacuum therapy; ^2^ FS-EVT: fistula sponge–endoluminal vacuum therapy.

**Table 3:** Overview of mortality.

	cEVT ^1^ (N = 47)	FS-EVT ^2^ (N = 24)	*p*-Value
Treatment-associated 30-day mortality [N] (%)	2 (4.3)	1 (4.0)	>0.99
30-day mortality total [N] (%)	10 (21.3)	5 (20.8)	0.08
6-month mortality total [N] (%)	1 (2.1)	2 (8.3)	0.26
Completed 12-month follow-up	36 (76.6)	17 (70.8)	0.77

^1^ cEVT: conventional endoluminal vacuum therapy; ^2^ FS-EVT: fistula sponge–endoluminal vacuum therapy.

## Data Availability

The datasets used and/or analyzed during the current study are available from the corresponding author on reasonable request.

## References

[1] Abbas G., Schuchert M.J., Pettiford B.L., Pennathur A., Landreneau J., Landreneau J., Luketich J.D., Landreneau R.J. (2009). Contemporaneous management of esophageal perforation. Surgery.

[2] Brinster C.J., Singhal S., Lee L., Marshall M.B., Kaiser L.R., Kucharczuk J.C. (2004). Evolving options in the management of esophageal perforation. Ann. Thorac. Surg..

[3] Chirica M., Champault A., Dray X., Sulpice L., Munoz-Bongrand N., Sarfati E., Cattan P. (2010). Esophageal perforations. J. Visc. Surg..

[4] Erdogan A., Gurses G., Keskin H., Demircan A. (2007). The sealing effect of a fibrin tissue patch on the esophageal perforation area in primary repair. World J. Surg..

[5] Hermansson M., Johansson J., Gudbjartsson T., Hambreus G., Jonsson P., Lillo-Gil R., Smedh U., Zilling T. (2010). Esophageal perforation in South of Sweden: Results of surgical treatment in 125 consecutive patients. BMC Surg..

[6] Jones W.G., Ginsberg R.J. (1992). Esophageal perforation: A continuing challenge. Ann. Thorac. Surg..

[7] de Schipper J.P., Pull ter Gunne A.F., Oostvogel H.J., van Laarhoven C.J. (2009). Spontaneous rupture of the oesophagus: Boerhaave’s syndrome in 2008. Literature review and treatment algorithm. Dig. Surg..

[8] Braghetto I., Rodriguez A., Csendes A., Korn O. (2005). An update on esophageal perforation. Rev. Med. Chil..

[9] Eroglu A., Turkyilmaz A., Aydin Y., Yekeler E., Karaoglanoglu N. (2009). Current management of esophageal perforation: 20 years experience. Dis. Esophagus.

[10] Gupta N.M., Kaman L. (2004). Personal management of 57 consecutive patients with esophageal perforation. Am. J. Surg..

[11] Okten I., Cangir A.K., Ozdemir N., Kavukcu S., Akay H., Yavuzer S. (2001). Management of esophageal perforation. Surg. Today.

[12] Vogel S.B., Rout W.R., Martin T.D., Abbitt P.L. (2005). Esophageal perforation in adults: Aggressive, conservative treatment lowers morbidity and mortality. Ann. Surg..

[13] Boshier P.R., Anderson O., Hanna G.B. (2011). Transthoracic versus transhiatal esophagectomy for the treatment of esophagogastric cancer: A meta-analysis. Ann. Surg..

[14] Rutegard M., Lagergren P., Rouvelas I., Lagergren J. (2012). Intrathoracic anastomotic leakage and mortality after esophageal cancer resection: A population-based study. Ann. Surg. Oncol..

[15] Escofet X., Manjunath A., Twine C., Havard T.J., Clark G.W., Lewis W.G. (2010). Prevalence and outcome of esophagogastric anastomotic leak after esophagectomy in a UK regional cancer network. Dis. Esophagus.

[16] Svendsen L.B., Jensen L.S., Holm J., Kofoed S.C., Pilegaard H., Preisler L., Vinbaek M., Brandt B., Svendsen M.B., Danish Oesophagus G.E.J. (2013). Differences in the pattern of anastomotic leakage after oesophagectomy in two high-volume centres. Dan. Med. J..

[17] Page R.D., Shackcloth M.J., Russell G.N., Pennefather S.H. (2005). Surgical treatment of anastomotic leaks after oesophagectomy. Eur. J. Cardio-Thorac. Surg. Off. J. Eur. Assoc. Cardio-Thorac. Surg..

[18] Bohm G., Mossdorf A., Klink C., Klinge U., Jansen M., Schumpelick V., Truong S. (2010). Treatment algorithm for postoperative upper gastrointestinal fistulas and leaks using combined vicryl plug and fibrin glue. Endoscopy.

[19] Hampe J., Schniewind B., Both M., Fritscher-Ravens A. (2010). Use of a NOTES closure device for full-thickness suturing of a postoperative anastomotic esophageal leakage. Endoscopy.

[20] Brangewitz M., Voigtlander T., Helfritz F.A., Lankisch T.O., Winkler M., Klempnauer J., Manns M.P., Schneider A.S., Wedemeyer J. (2013). Endoscopic closure of esophageal intrathoracic leaks: Stent versus endoscopic vacuum-assisted closure, a retrospective analysis. Endoscopy.

[21] Schniewind B., Schafmayer C., Voehrs G., Egberts J., von Schoenfels W., Rose T., Kurdow R., Arlt A., Ellrichmann M., Jurgensen C. (2013). Endoscopic endoluminal vacuum therapy is superior to other regimens in managing anastomotic leakage after esophagectomy: A comparative retrospective study. Surg. Endosc..

[22] Loske G., Rucktaeschel F., Schorsch T., Moenkemueller K., Mueller C.T. (2019). Endoscopic negative pressure therapy (ENPT) for duodenal leakage—Novel repair technique using open-pore film (OFD) and polyurethane-foam drainages (OPD). Endosc. Int. Open.

[23] Ahrens M., Schulte T., Egberts J., Schafmayer C., Hampe J., Fritscher-Ravens A., Broering D.C., Schniewind B. (2010). Drainage of esophageal leakage using endoscopic vacuum therapy: A prospective pilot study. Endoscopy.

[24] Pennathur A., Luketich J.D. (2008). Resection for esophageal cancer: Strategies for optimal management. Ann. Thorac. Surg..

[25] Schmidt H., Manegold B.C., Stuker D., Grund K.E. (2001). Anastomotic insufficiencies of the esophagus--early surgical endoscopy and endoscopic therapy. Kongressbd Dtsch. Ges. Chir. Kongr..

[26] Leers J.M., Vivaldi C., Schafer H., Bludau M., Brabender J., Lurje G., Herbold T., Holscher A.H., Metzger R. (2009). Endoscopic therapy for esophageal perforation or anastomotic leak with a self-expandable metallic stent. Surg. Endosc..

[27] Law R., Prabhu A., Fujii-Lau L., Shannon C., Singh S. (2018). Stent migration following endoscopic suture fixation of esophageal self-expandable metal stents: A systematic review and meta-analysis. Surg. Endosc..

[28] van Halsema E.E., Rauws E.A., Fockens P., van Hooft J.E. (2015). Self-expandable metal stents for malignant gastric outlet obstruction: A pooled analysis of prospective literature. World J. Gastroenterol..

[29] Heits N., Stapel L., Reichert B., Schafmayer C., Schniewind B., Becker T., Hampe J., Egberts J.H. (2014). Endoscopic endoluminal vacuum therapy in esophageal perforation. Ann. Thorac. Surg..

[30] Kuehn F., Schiffmann L., Rau B.M., Klar E. (2012). Surgical endoscopic vacuum therapy for anastomotic leakage and perforation of the upper gastrointestinal tract. J. Gastrointest. Surg. Off. J. Soc. Surg. Aliment. Tract..

[31] Loske G., Schorsch T., Rucktaeschel F., Schulze W., Riefel B., van Ackeren V., Mueller C.T. (2018). Open-pore film drainage (OFD): A new multipurpose tool for endoscopic negative pressure therapy (ENPT). Endosc. Int. Open.

[32] Wichmann D., Stuker D., Schempf U., Werner C.R., Steger V., Konigsrainer A., Schweizer U., Archid R. (2020). Endoscopic negative pressure therapy with open-pore film drainage and open-pore polyurethane sponge drainage for iatrogenic perforation of the esophagus. Endoscopy.

[33] Kaczmarek D.J., Heling D.J., Gonzalez-Carmona M.A., Strassburg C.P., Branchi V., Matthaei H., Kalff J., Manekeller S., Glowka T.R., Weismuller T.J. (2021). Management of post-operative pancreatic fistulas following Longmire-Traverso pylorus-preserving pancreatoduodenectomy by endoscopic vacuum-assisted closure therapy. BMC Gastroenterol..

[34] Wulfert C.H., Muller C.T., Abdel-Kawi A.F., Schulze W., Schmidt-Seithe H., Borstelmann S., Loske G. (2020). Intrauterine negative-pressure therapy (IU-NPT) to treat peritonitis after caesarean section. Innov. Surg. Sci..

[35] Loske G., Schorsch T., Kiesow R.U., Muller C.T. (2017). First report of urinary endoscopic vacuum therapy: For large bladder defect after abdomino-perineal excision of the rectum. Video paper. Der Chir. Z. Fur Alle Geb. Der Oper. Medizen.

[36] Richter F., Hendricks A., Schniewind B., Hampe J., Heits N., von Schonfels W., Reichert B., Eberle K., Ellrichmann M., Baumann P. (2022). Eso-Sponge(R) for anastomotic leakage after oesophageal resection or perforation: Outcomes from a national, prospective multicentre registry. BJS Open.

[37] Loske G., Schorsch T., Muller C. (2010). Endoscopic vacuum sponge therapy for esophageal defects. Surg. Endosc..

[38] Bludau M., Holscher A.H., Herbold T., Leers J.M., Gutschow C., Fuchs H., Schroder W. (2014). Management of upper intestinal leaks using an endoscopic vacuum-assisted closure system (E-VAC). Surg. Endosc..

[39] Laukoetter M.G., Mennigen R., Neumann P.A., Dhayat S., Horst G., Palmes D., Senninger N., Vowinkel T. (2017). Successful closure of defects in the upper gastrointestinal tract by endoscopic vacuum therapy (EVT): A prospective cohort study. Surg. Endosc..

[40] Moschler O., Nies C., Mueller M.K. (2015). Endoscopic vacuum therapy for esophageal perforations and leakages. Endosc. Int. Open.

[41] Pournaras D.J., Hardwick R.H., Safranek P.M., Sujendran V., Bennett J., Macaulay G.D., Hindmarsh A. (2018). Endoluminal Vacuum Therapy (E-Vac): A Treatment Option in Oesophagogastric Surgery. World J. Surg..

[42] Schorsch T., Muller C., Loske G. (2013). Endoscopic vacuum therapy of anastomotic leakage and iatrogenic perforation in the esophagus. Surg. Endosc..

[43] Jung C.F.M., Muller-Dornieden A., Gaedcke J., Kunsch S., Gromski M.A., Biggemann L., Seif Amir Hosseini A., Ghadimi M., Ellenrieder V., Wedi E. (2021). Impact of Endoscopic Vacuum Therapy with Low Negative Pressure for Esophageal Perforations and Postoperative Anastomotic Esophageal Leaks. Digestion.

[44] Loske G., Muller C.T. (2019). Tips and tricks for endoscopic negative pressure therapy. Der Chir. Z. Fur Alle Geb. Der Oper. Medizen.

[45] Bludau M., Fuchs H.F., Herbold T., Maus M.K.H., Alakus H., Popp F., Leers J.M., Bruns C.J., Holscher A.H., Schroder W. (2018). Results of endoscopic vacuum-assisted closure device for treatment of upper GI leaks. Surg. Endosc..

[46] Binda C., Jung C.F.M., Fabbri S., Giuffrida P., Sbrancia M., Coluccio C., Gibiino G., Fabbri C. (2023). Endoscopic Management of Postoperative Esophageal and Upper GI Defects-A Narrative Review. Medicina.

[47] Laopeamthong I., Akethanin T., Kasetsermwiriya W., Techapongsatorn S., Tansawet A. (2022). Vacuum Therapy and Internal Drainage as the First-Line Endoscopic Treatment for Post-Bariatric Leaks: A Systematic Review and Meta-Analysis. Visc. Med..

[48] Jung C.F.M., Hallit R., Muller-Dornieden A., Calmels M., Goere D., Chaput U., Camus M., Gonzalez J.M., Barthet M., Jacques J. (2022). Endoscopic internal drainage and low negative-pressure endoscopic vacuum therapy for anastomotic leaks after oncologic upper gastrointestinal surgery. Endoscopy.

[49] Lange J., Dormann A., Bulian D.R., Hugle U., Eisenberger C.F., Heiss M.M. (2021). VACStent: Combining the benefits of endoscopic vacuum therapy and covered stents for upper gastrointestinal tract leakage. Endosc. Int. Open.

[50] Lange J., Knievel J., Wichmann D., Kahler G., Wiedbrauck F., Hellmich T., Kandler M., Bernhardt J., Scholz D., Beyna T. (2023). Clinical implantation of 92 VACStents in the upper gastrointestinal tract of 50 patients-applicability and safety analysis of an innovative endoscopic concept. Front. Surg..

[51] Loske G., Schorsch T., Muller C.T. (2017). Prevention of reflux after esophagectomy with endoscopic negative pressure therapy using a new double-lumen open-pore film drainage with an intestinal feeding tube. Endoscopy.

